# Lightweight Convolutional Neural Network with Efficient Channel Attention Mechanism for Real-Time Facial Emotion Recognition in Embedded Systems

**DOI:** 10.3390/s25237264

**Published:** 2025-11-28

**Authors:** Juan A. Ramirez-Quintana, Jesus J. Muñoz-Pacheco, Graciela Ramirez-Alonso, Jesus A. Medrano-Hermosillo, Alma D. Corral-Saenz

**Affiliations:** 1 Graduate Studies and Research Division, Tecnológico Nacional de México/I.T. Chihuahua, Chihuahua 31200, Mexico; l23061083@chihuahua.tecnm.mx (J.J.M.-P.); jesus.mh@chihuahua.tecnm.mx (J.A.M.-H.); alma.cs@chihuahua.tecnm.mx (A.D.C.-S.); 2 Faculty of Engineering, Universidad Autonoma de Chihuahua Campus II, Chihuahua 31125, Mexico; galonso@uach.mx

**Keywords:** deep learning, real-time processing, emotion recognition, facial expression recognition

## Abstract

This paper presents a novel deep neural network for real-time emotion recognition based on facial expression measurement, optimized for low computational complexity, called Lightweight Expression Recognition Network (LiExNet). The LiExNet architecture comprises only 42,000 parameters and integrates convolutional layers, depthwise convolutional layers, an efficient channel attention mechanism, and fully connected layers. The network was trained and evaluated on three widely used datasets (CK+, KDEF, and FER2013) and a custom dataset, EMOTION-ITCH. This dataset comprises facial expressions from both industrial workers and non-workers, enabling the study of emotional responses to occupational stress. Experimental results demonstrate that LiExNet achieves high recognition performance with minimal computational resources, reaching 99.5% accuracy on CK+, 88.2% on KDEF, 79.2% on FER2013, and 96% on EMOTION-ITCH. In addition, LiExNet supports real-time inference on embedded systems, requiring only 0.03 MB of memory and 1.38 GFLOPs of computational power. Comparative evaluations show that among real-time methods, LiExNet achieves the best results, ranking first on the CK+ and KDEF datasets, and second on FER2013, demonstrating consistent performance across these datasets. These results position LiExNet as a practical and robust alternative for real-time emotion monitoring and emotional dissonance assessment in occupational settings, including hardware-constrained and embedded environments.

## 1. Introduction

Emotion recognition using artificial intelligence relies on computational methods that analyze human emotional states from various input sources. These include physical signals such as voice, facial expressions, and body posture, as well as physiological signals such as heart rate and brain activity, which are captured through microphones, cameras, and biosensors [1]. Physiological signals such as electroencephalography (EEG) have shown promising results in emotion recognition because they can capture affective states through direct measurements of brain activity or by combining them with other modalities. Recent studies highlight the importance of spatiotemporal representation fusion to model the dynamic evolution of neural activation patterns associated with emotional states [2]. Similarly, Liu et al. [3] examined emotional recovery by analyzing variations in EEG frequency-band activity when individuals were exposed to natural sound environments, demonstrating the relevance of neural signals for monitoring affective responses. However, the acquisition of EEG and other physiological signals requires dedicated instrumentation, calibration, and noise-controlled environments, which restrict their deployment in everyday settings, affective computing frameworks, and real-world evaluations of occupational dissonance [4]. Considering this, facial expression recognition has gained significant attention due to the ubiquity of imaging sensors and their non-intrusive nature, providing a widely applicable and accessible channel for emotion analysis. Although facial expressions can be consciously controlled or feigned, microexpressions provide reliable signals of genuine affective states [5,6,7]. Recent studies provide empirical evidence that image sensors, combined with image processing and neural network models, are effective for detecting facial expressions and microexpressions, enabling fast, unobtrusive affective monitoring in real-world environments [8]. Such approaches are being applied across various domains, including occupational health and emotional dissonance monitoring [9,10], as well as adaptive learning, automotive safety, and customer service systems [5,7].

The literature reports several real-time facial emotion recognition (FER) methods developed using image datasets that serve as benchmarks for evaluating algorithms that integrate computer vision and artificial intelligence with camera and sensor systems. These datasets capture facial expressions from individuals in both controlled laboratory conditions and real-world scenarios. Among the most widely used and well-established datasets are the Extended Cohn–Kanade Dataset (CK+) [11], the Facial Expression Recognition 2013 (FER2013) [12], and the Karolinska Directed Emotional Faces (KDEF) [13].

For example, Yang et al. proposed an algorithm that interprets facial Action Units (expressions) to find emotions with the Facial Action Coding System (FACS) and a Convolutional Neural Network (CNN) [14]. They evaluated their algorithm in real time using the CK+ dataset and reported an average accuracy of 92.4%. The models proposed in [15,16,17] are CNN-based architectures for real-time FER applications that achieved accuracies of 95.65% [15], 98.5% [16], and 98% [17] using the CK+ dataset. Happy and Routray [18] proposed a method based on Local Binary Patterns (LBP) and Support Vector Machine (SVM) for real-time FER, achieving an accuracy of 94% on the CK+ dataset. Gupta et al. designed a system to assess student engagement in real-time online learning environments through FER [19]. Their approach employed Inception-V3, VGG-19, and ResNet-50, trained on the CK+ dataset. Among these networks, ResNet-50 achieved the highest accuracy (92.32%), followed by VGG-19 (90.14%) and Inception-V3 (89.11%). The models of [20,21] are CNN architectures trained and evaluated on FER2013, achieving accuracies of 75.1% [20] and 63.2% [21]. Sholikah et al. developed a real-time mobile application based on VGG16 to support children with autism [22]. The application achieved an accuracy of 91% on FER2013, demonstrating its effectiveness in understanding emotional learning in autistic children. Shesu et al. introduced LEmo, a method for facial emotion categorization that is resistant to adversarial and distractor attacks while maintaining low computational requirements, making it suitable for near real-time applications [23]. LEmo achieved an accuracy of 97.86% on CK+ and 85% on KDEF. Finally, Hussain et al. proposed a method based on the VGG16 architecture and the KDEF dataset, reporting an accuracy of 88% [24].

Numerous approaches have explored methods based on CNN, traditional machine learning, or transformers for FER, which are also trained and evaluated on FER2013, CK+, or KDEF, achieving competitive performance in facial emotion recognition. Although these methods achieve strong results with only facial expressions, they are generally not designed for real-time processing or deployment on embedded or ubiquitous systems. Nevertheless, they provide valuable baselines and design references for the development of lightweight approaches that enable real-time facial expression recognition on embedded platforms. For example, Ullah et al. [25] proposed a system composed of Canonical Correlation Analysis, a CNN, and a Long Short-Term Memory (LSTM) that achieved an accuracy of 91.42% on the CK+ dataset. The models in [26,27,28] are CNNs that reported accuracies of 99.25%, 93.24%, and 98.38%, respectively. Other CNN-based approaches were trained and evaluated on the CK+ and FER2013 datasets [29,30,31,32]. The CNN models proposed in [29,30] achieved competitive results: [29] reported accuracies of 94% on CK+ and 73% on FER2013, while [30] reported accuracies of 96% on CK+ and 71% on FER2013. The method in [31] integrates CNN, LBP, and an attention module, achieving accuracies of 98.9% on CK+ and 75.82% on FER2013. The algorithm in [32] is a CNN that incorporates deep reinforcement learning, reaching accuracies of 94.1% on CK+ and 72.1% on FER2013. The model presented in [33] is a CNN architecture that achieved an accuracy of 88.56% on FER2013. Haider et al. [34] presented a hybrid model that extracts features using a CNN and classifies them with an SVM, achieving 74.64% accuracy on FER2013. Akhand et al. [35] proposed a CNN-based pipeline developed through an evaluation of several pre-trained architectures, including VGG16/19, ResNet-18/50/152, Inception-V3, and DenseNet-161. Their experiments on the KDEF and JAFFE datasets using 10-fold cross-validation showed that DenseNet-161 performed best, achieving 96.51% accuracy on KDEF and 99.52% on JAFFE.

Finally, Khan et al. [36] introduced an ensemble framework combining ResNet-50 and Inception-V3 within an attention-enhanced deep ensemble network, which reached 99.3% accuracy on KDEF and 78.6% on FER2013 under 10-fold cross-validation. Sen et al. [37] proposed a method that combines LBP with an SVM classifier, achieving an accuracy of 91.11% on the CK+ dataset. Kumar et al. [38] introduced a model that integrates the wavelet transform, multivariate analysis, and a Fuzzy SVM, which achieved 98.9% accuracy on CK+. More recently, Nawaz et al. [39] proposed a transformer-based architecture that dynamically focuses on the most informative facial regions via adaptive attention mechanisms. Their model also implements dynamic token pruning to eliminate less relevant tokens during intermediate processing stages. This transformer-based approach achieved state-of-the-art performance, with 99.67% accuracy on FER2013 and 99.52% on CK+.

The reviews presented in [1,5], along with our literature analysis, highlight the continuous progress in facial emotion recognition using transformer-based and CNN-based approaches, such as Vision Transformers (ViT), attention mechanisms, Inception, VGG, ResNet, MobileNet, and Xception. Although these methods achieve high accuracy, they typically require large training datasets and rely on architectures with millions of parameters, as well as substantial computational resources, which limits their deployment on embedded platforms and other low-power, resource-constrained systems.

Other approaches aim to reduce model complexity, but they often report lower accuracy or are validated on a limited number of datasets, making it difficult to assess their generalization and robustness in real-world sensor-based environments. In addition, research addressing occupational environments remains scarce, despite their relevance for applications such as stress measurement and safety monitoring. These limitations can be addressed by developing novel architectures that achieve competitive recognition performance while maintaining low computational cost, thereby enabling real-time deployment on embedded platforms. Therefore, this paper proposes the Lightweight Expression Recognition Network (LiExNet), a novel neural architecture that achieves consistent learning and generalization across multiple datasets with heterogeneous facial information—including frontal faces captured under controlled conditions as well as multi-angle faces in unconstrained environments—attaining top accuracy while remaining suitable for real-time deployment on embedded and ubiquitous image-sensor systems [40]. The main contributions of LiExNet are:

- Present a lightweight deep neural network with only 42,000 parameters, combining standard and depthwise separable convolutions with an efficient channel attention mechanism, specifically designed to enable real-time inference on embedded sensor platforms with minimal computational resources.
- Provide an extensive evaluation on three widely used facial expression datasets (CK+, KDEF, FER2013) and on EMOTION-ITCH, a novel dataset focused on emotion recognition under occupational stress conditions.
- Achieves state-of-the-art accuracy (99.5% on CK+) with only 86 MFLOPs, a 0.17 MB model memory footprint, a 2 MB peak inference memory, and real-time performance above 530 FPS on an Jetson TX2 (NVIDIA Corporation, Santa Clara, CA, USA).
- Introduce EMOTION-ITCH as a new public dataset that includes facial expressions from both working and non-working participants, supporting the measure and analysis of emotional responses in industrial environments and enabling fine-tuning experiments that demonstrate improvements when occupational stress information is incorporated.

The rest of the paper is organized as follows: Section 2 presents the LiExNet model, Section 3 describes the datasets used for experiments, and Section 4 presents the results. Finally, Section 5 provides the conclusions.

## 2. Lightweight Expression Recognition Network

According to the state-of-the-art analysis, the following conclusions can be made:

- Most FER models are based on CNN architectures, among which VGG16 is the most widely used.
- Real-time FER models that achieve competitive performance are typically based on traditional CNN architectures [16,22], CNNs enhanced with attention mechanisms [17], or hybrid approaches that incorporate facial landmarks [23]. Among these, MobileNet is the most widely adopted architecture for developing real-time embedded applications.
- The FER models that achieve the highest performance are either very deep traditional CNN architectures [35,36] or transformer-based models [24]. These models are focused on improving performance, but are not designed for embedded or ubiquitous systems.

Based on these observations, LiExNet was designed as a compact CNN architecture enhanced with an efficient channel attention mechanism to better focus on expression and microexpression features.

**Figure 1:**
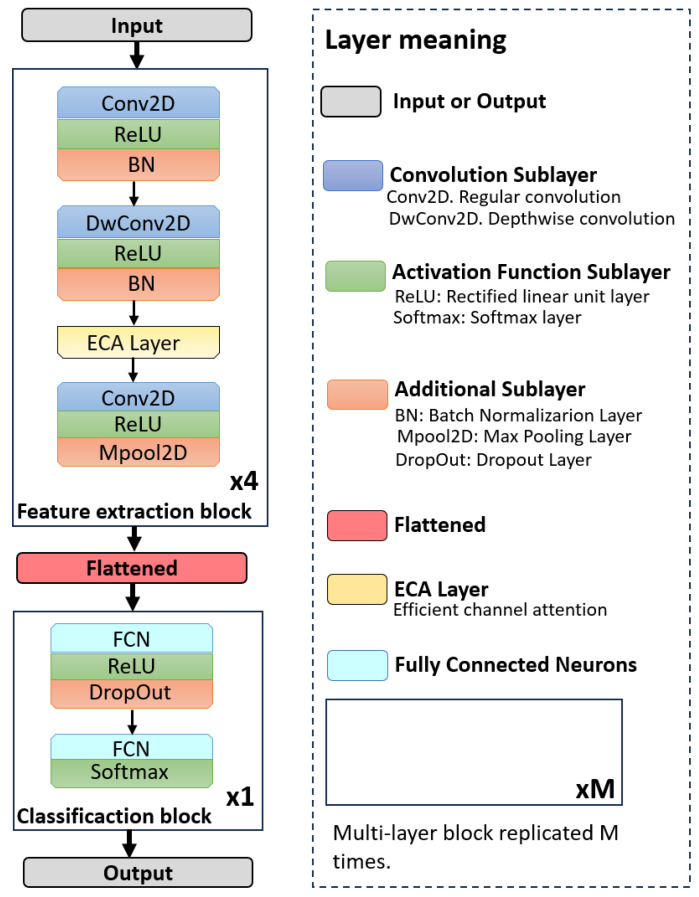
LiExNet architecture.

Figure 1 presents the overall architecture of LiExNet, organized into the following stages: input layer, feature extraction block, flattening layer, classification block, and output layer.

The input layer receives a facial expression image, either sourced from benchmark datasets or captured in real time through a camera or other imaging sensor. The feature extraction stage comprises four blocks, each containing an efficient channel attention (ECA) module and convolutional layers equipped with ReLU activations, batch normalization, and max pooling. The output of this block is a set of feature representations, which are subsequently converted into a one-dimensional feature vector by the flatten layer. The classification block then processes this feature vector to determine the corresponding emotion class conveyed by the input image. The output layer produces the final predicted emotion, which can be utilized in real-time applications. The following subsections provide a detailed description of LiExNet.

### 2.1. Input

The input to LiExNet is a color image $I{\left(m,n\right)}^{RGB}$ in 8-bit integer format, which is then transformed into a tensor $X_{r}\left(m,n\right)$ in float format, where (m,n) denotes the pixel position and *r* is the depth index. This tensor has values scaled to the range [0,1] and dimensions of 128×128 with a depth of 3, where r∈{1,2,3}.

### 2.2. Feature Extraction Blocks

The feature extraction stage consists of four sequential blocks that share the same architecture.

Each block begins with a standard convolutional sublayer to find local abstract features, mathematically defined as:

(1) $$\begin{array}{cc} C_{l,r}\left(m,n\right) & =f(W_{l}\left(m,n\right)*Y_{l-1,r}\left(m,n\right)+\beta_{l}),\\ l & =\{1,5,9,13\},\;r\in \{1,\ldots ,R\} \end{array}$$

where *l* is the layer index, *R* is the depth, $W_{l}\left(m,n\right)$ represents the weights, $Y_{l-1,r}\left(m,n\right)$ is the output of the previous layer, and $\beta_{l,r}$ is the bias term. The activation function f(·) is the Rectified Linear Unit (ReLU), which helps prevent vanishing gradients. The input of the first block is the tensor $Y_{0,r}\left(m,n\right)=X_{r}\left(m,n\right)$. The subsequent sublayer is batch normalization, denoted as g[·], and defined as $Y_{l,r}\left(m,n\right)=g\left[C_{l,r}\left(m,n\right)\right]$. The ReLU and batch normalization operations are described in [41]. The use of batch normalization after ReLU achieves faster convergence than other possible sublayer orderings within the block.

The next layer in each block is a depthwise convolution used for parameter reduction, defined as:

(2) $$\begin{array}{cc} C_{l,r}\left(m,n\right) & =f\left(W_{l,r}\left(m,n\right)*Y_{l,r}+\beta_{l,r}\right),\\ l & =\{2,6,10,14\},\;r\in \{1,\ldots ,R\} \end{array}$$

where $W_{l,r}\left(m,n\right)$ are the weights for channel *r*. As in the previous layer, the activation function f(·) is ReLU, followed by batch normalization g[·], defined as $Y_{l,r}\left(m,n\right)=g\left[C_{l,r}\left(m,n\right)\right]$.

The next layer is an efficient channel attention (ECA) module, a lightweight attention mechanism designed to emphasize the channels that carry the most relevant features by incorporating channel-wise attention with minimal computational overhead. This module consists of global average pooling, a one-dimensional convolution over channels, and channel-wise recalibration. The global average pooling operation, denoted as $Y_{l,r}\left(m,n\right)=\rho \left[C_{l,r}\left(m,n\right)\right]$, is described in [41], and the one-dimensional convolution is expressed as follows:

(3) $$\begin{array}{cc} C_{l,m}\left(n\right) & =f(W_{l}\left(n\right)*Y_{l-1,r,m}\left(n\right)+\beta_{l}),\\ l & =\{3,7,11,15\},\;r\in \{1,\ldots ,R\} \end{array}$$

where $Y_{l-1,m}\left(n\right)$ represents a column from the previous layer, and the activation function f(·) is the sigmoid function. The following equation determines the kernel size:

(4) $$\begin{array}{c} k=\psi \left(M\right)={\left|\frac{\log_{2}\left(M\right)}{\gamma }+f\right|}_{\mathrm{odd}} \end{array}$$

where γ and *f* are hyperparameters, experimentally set to γ=2 and f=1. Channel recalibration estimates the importance of each channel by reweighting them as follows:

(5) $$\begin{array}{cc} Y_{l,m}\left(n\right) & =\omega_{m}C_{l,m}\left(n\right), \end{array}$$

where $\omega_{m}$ is the scalar weight assigned to each $C_{l,m}\left(n\right)$.

The final layer of each block is a standard convolution used to extract local features from channels identified as most relevant. This layer is defined in Equation (1), where l∈{4,8,12,16} and r=1,…,R. The activation function f(·) is ReLU, followed by a max pooling operation with a window size of 2×2, represented as $Y_{l,r}\left(m,n\right)=h\left[C_{l,r}\left(m,n\right)\right]$. Max pooling is used to reduce overfitting and computational cost, as described in [41].

The convolution and depthwise layers follow the initial design principle of the MobileNet architecture, whereas VGG16 inspires the hierarchical stacked block structure. Depthwise convolutions significantly reduce FLOPs and the number of parameters, while the ECA module introduces channel-wise attention at minimal computational cost. The number of filters increases progressively across the four convolutional blocks to expand the feature space, whereas each depthwise layer maintains a single kernel per channel. The increase in network depth was determined empirically, based on the hypothesis that increasing depth across the four convolutional blocks allows the model to capture progressively more abstract and discriminative facial representations, thereby enhancing overall recognition performance.

The first feature extraction block uses 24 convolutional kernels (l={1,4}) to separate the face from the background, reducing the amount of irrelevant information captured by the sensor. The second block uses 48 kernels (l={5,8}) and emphasizes higher-level facial components that are essential for robust recognition under varying acquisition conditions. The third block uses 72 kernels (l={9,12}) and focuses on expression-related regions (Action Units), including the cheeks, chin, mouth, and the T-shaped area between the eyes and nose. The fourth block uses 96 kernels (l={13,16}) and captures subtle microexpression features, which contribute to the interpretation of authentic emotional states.

### 2.3. Flattened Layer

Layer l=10 transforms the output $Y_{9,r}\left(m,n\right)$ into a feature vector $Z_{10}\left(k\right)$, where k=M·N·R, with *M* denoting the number of rows and *N* the number of columns.

### 2.4. Classification Block

The classification block consists of two FCN layers. The first layer in this block, l=11, is defined as:

(6) $$\begin{array}{cc} C_{11,r}\left(k\right) & =f\left(W_{11,r}\left(k\right)\cdot Z_{10}\left(k\right)+\beta_{l,r}\right),\\ l & =11,\;r=1,\ldots ,256 \end{array}$$

The activation function f(·) for this layer is the ReLU function. Subsequently, a dropout layer is applied to remove 30% of the neurons during each training epoch [42]. The output of this layer is denoted as $Y_{11}\left(m,n\right)$. The next layer is an FCN, defined as follows:

(7) $$\begin{array}{cc} y_{12,r}\left(k\right) & =f\left(W_{12,r}\left(k\right)\cdot Y_{11,r}\left(m,n\right)+\beta_{12,r}\right),\\ l & =12,\;r=1,\ldots ,D \end{array}$$

where *D* is the number of classes. The maximum value of the output layer, $y_{12,r}\left(k\right)$, represents the predicted emotion label *y* obtained by LiExNet. The value of *y* is compared with the desired label value *l* during training.

### 2.5. Output Layer

The output layer encodes *y* as data that represents the detected emotion. This information can be transmitted to other embedded systems or servers for further processing.

## 3. Datasets

This section describes the datasets utilized for this research. The datasets include CK+, KDEF, and FER2013, which are well-known in the literature, and each contains seven emotional categories. Therefore, the output size of LiExNet should be set to D=7. Additionally, this work introduces another dataset, EMOTION-ITCH, with three categories, yielding an output size of D=3.

### 3.1. Extended Cohn-Kanade Dataset (CK+)

**Figure 2:**
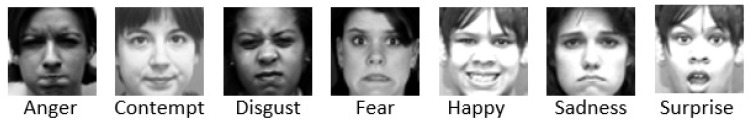
CK+ expression examples.

The CK+ dataset consists of 593 frontal facial expression images captured at 30 frames per second, with resolutions of 640×490 or 640×480 pixels. The images were taken under controlled lighting conditions in a laboratory. These recordings were collected from a diverse group of subjects aged 18 to 50, representing a range of genders and ethnic backgrounds. Each video is labeled with one of the following emotional expressions: anger (AN), disgust (DI), fear (FE), happiness (HA), sadness (SA), surprise (SU), and contempt (CO) [11]. Figure 2 displays an example for each emotion class in the dataset.

### 3.2. Karolinska Directed Emotional Faces (KDEF)

**Figure 3:**
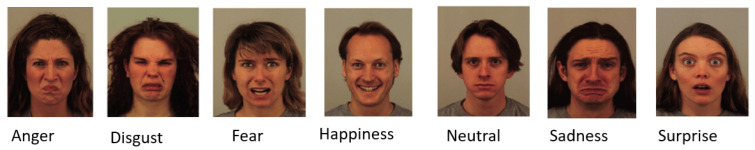
KDEF expression examples.

The KDEF dataset was developed by the Department of Clinical Neuroscience, Psychology Section at the Karolinska Institute in Stockholm, Sweden. It contains 49,000 images of 70 subjects (35 male and 35 female), aged 20–30 years. The acquisition system was placed in a studio with controlled lighting and a uniform background and consisted of a digital camera placed in a trifocal configuration ($0^{\circ }$ frontal, $30^{\circ }$ left, and $60^{\circ }$ right [13]) with constant conditions of distance, focus, and position of the subject with respect to the camera and without occlusions. The images are in RGB format, with a resolution of 562×762. The images were annotated into seven emotional expressions: AN, DI, HA, SA, SU, Afraid (AF, also referred to as Fear), and Neutral (NE). Figure 3 displays one example of each expression class in the dataset.

### 3.3. Facial Expression Recognition 2013 (FER2013)

**Figure 4:**
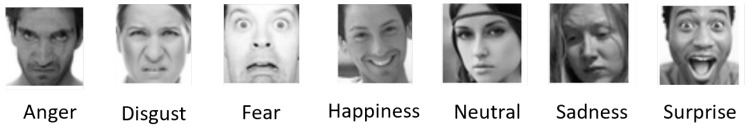
FER2013 expression examples.

FER2013 is a dataset comprising 35,887 images collected from the web. Therefore, the images were acquired from different conventional cameras under various lighting conditions, occlusions, and poses. All images were preprocessed to grayscale at a resolution of 48×48. The dataset includes seven emotion classes: AN, DI, FE, HA, SA, SU, and NE. In this work, a processed version of FER2013 is used [12] because the original FER2013 dataset has class imbalance and quality issues. Figure 4 shows one example of each expression class in the dataset.

### 3.4. Custom Dataset—EMOTION-ITCH

The EMOTION-ITCH dataset was developed at the Instituto Tecnológico de Chihuahua (ITCH) to analyze emotional responses and occupational stress using multimodal sensor data. The dataset consists of 587 facial expression images acquired using a conventional smartphone camera at a resolution of 1920×1080, 492 speech audio recordings captured with a smartphone microphone in MP3 format, and 260 EEG recordings obtained through gold cup electrodes and the OpenBCI device. A total of 30 non-actor participants were recruited, and their emotions were elicited and categorized into three classes: neutral, positive, and negative. Emotions were grouped into positive, neutral, and negative categories following the affective valence theory and the circumplex model [43]. Additionally, grouping into three affective classes improves class balance and supports reliable modeling in real-time embedded environments, where lightweight architectures must avoid class fragmentation [44].

For each participant, fifteen facial images were collected (five per emotional category). Emotional ground-truth labels were obtained using the Self-Assessment Manikin (SAM), a validated and language-independent instrument widely used in affective computing and compatible with positive, neutral, and negative affective groupings [45,46]. The SAM scores were analyzed by discretizing the valence dimension into three classes: values >6 were labeled as positive, values <4 as negative, and values in the range 4–6 as neutral. SAM was administered twelve times per participant during a single session [47]. Additionally, structured interviews were conducted by five technicians involved in the development of EMOTION-ITCH.

**Figure 5:**
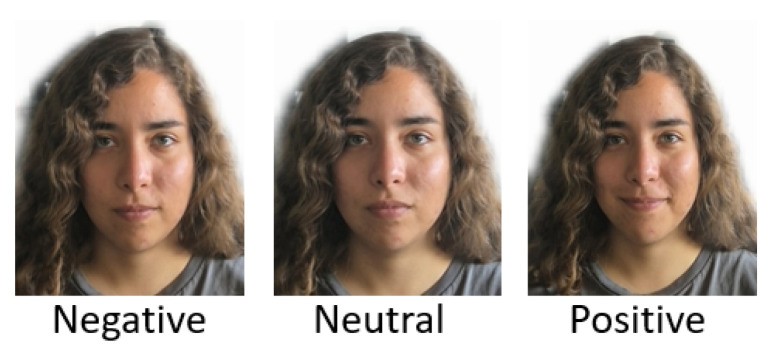
EMOTION-ITCH expression examples.

Among the 30 participants, 15 are employed in operational roles with high cognitive demands in a typical manufacturing plant located in the city of Chihuahua, while the remaining 15 are not currently working. This participant recruitment allows an analysis of differences in emotional responses between employed individuals with habitual work stress and those outside the workforce. Figure 5 shows one example for each emotion class in the dataset, and it is available at https://sites.google.com/view/laboratoriodps/download?authuser=0 (updated on 25 November 2025).

This dataset was selected for a fine-tuning experiment to investigate differences in facial expressions associated with emotions among employed and non-employed individuals.

## 4. Results

This section reports the results of six experiments conducted to evaluate the performance, consistency, and generalization of LiExNet: cross-validation and statistical significance, final performance assessment, Grad-CAM visualization, ablation study, comparison with state-of-the-art models, and fine-tuning on the EMOTION-ITCH dataset.

### 4.1. Cross-Validation and Statistical Significance

Cross-validation was used to assess generalization consistency, and statistical significance testing was used to confirm the reliability of LiExNet’s accuracy across the CK+, KDEF, and FER2013 datasets.

The cross-validation analysis was performed using a 5-fold scheme. This configuration was selected because, as reported in [48], it is more suitable than 10-fold cross-validation for datasets of the size of CK+, KDEF, and FER2013.

Table 1 presents the cross-validation results, where LiExNet achieves a mean accuracy of 99.3 ± 1.2 on the CK+ dataset, corresponding to a generalization range of 98.3% to 100%. For the KDEF dataset, the mean and standard deviation are 88.3 ± 0.75, indicating consistent generalization within an accuracy range of 87.55% to 89.05%. For the FER2013 dataset, the mean and standard deviation are 79.2 ± 0.9, indicating good generalization within an accuracy range of 78.3% to 80.1%.

The statistical significance analysis consisted of a linear variability study and a hypothesis test to compare the set of true test labels $L=\{l_{1},\ldots ,l_{T}\}$ from all datasets with the corresponding model outputs $Y=\{y_{1},\ldots ,y_{T}\}$, where *T* denotes the number of samples in the test set.

The linear variability study was performed using the Pearson correlation coefficient $\rho_{L,Y}$, which quantifies the strength and direction of the linear relationship between *L* and *Y* in each cross-validation partition [49]. The hypothesis test was conducted to determine whether the differences between *L* and *Y* are statistically significant, that is, whether they exhibit random variation or reflect a consistent relationship. The statistical significance was evaluated using the Wilcoxon signed-rank test, a nonparametric test for non-normal paired samples that assesses whether the difference between the two distributions is statistically significant [50].

**Figure 6:**
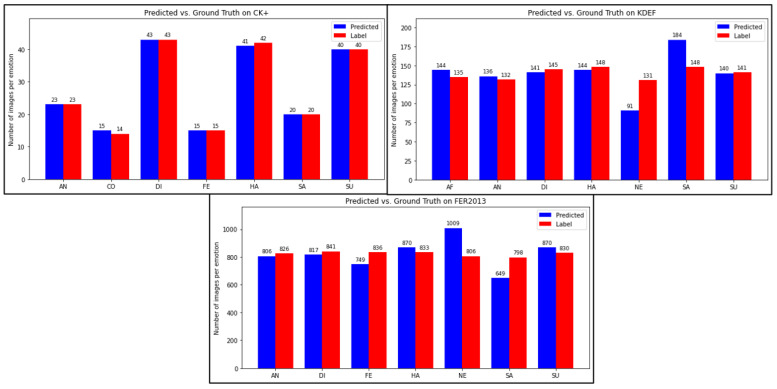
Predicted vs. Label with CK+, KDEF, and FER2013.

Table 2 presents the results of the Pearson correlation coefficient $\rho_{L,Y}$ and the Wilcoxon test statistics $h_{L,Y}$ and $p_{L,Y}$, while Figure 6 illustrates the histograms generated by grouping the true labels *L* and the predicted outputs *Y* from each fold.

In the case of CK+, the values of $\rho_{L,Y}$ and the Wilcoxon test indicate that *L* and *Y* are almost identical in all the folds. This behavior is illustrated in Figure 6, where the only observed difference is due to a single misclassified sample.

For the KDEF dataset, the $\rho_{L,Y}$ values are approximately 0.8, indicating a strong positive linear relationship between *L* and *Y*, implying that *Y* can be reliably estimated from *L* using a linear regression model. The Wilcoxon test indicates that the folds k=1,4,5 do not exhibit statistical significance, as *L* and *Y* show similar distributions. In contrast, the folds k=2,3 exhibit statistical significance, although their corresponding *p*-values suggest that the difference is minimal. This behavior can be attributed to specific cases in which LiExNet failed to correctly recognize certain facial expressions belonging to the NE and SA categories. For example, in Figure 6, noticeable differences can be observed between the NE and SA distributions.

In the case of FER2013, the $\rho_{L,Y}$ values are approximately 0.8, indicating a strong positive linear relationship between *L* and *Y*, showing that *Y* can be reliably approximated from *L* using a linear regression model. However, the Wilcoxon test reveals statistical significance across all folds. The corresponding *p*-values suggest that the folds k=3,4,5 include samples affected by uncontrolled imaging conditions, whereas the folds k=1,2 contain cases in which LiExNet failed to recognize certain facial expressions correctly. As shown in Figure 6, although the most significant errors again occur in the NE and SA categories, the misclassifications are more pronounced than on the KDEF dataset.

It is also noteworthy that although the average accuracies in cross-validation on FER2013 (79.2%) and KDEF (88.3%) differ by nearly 10%, the values of the Pearson correlation coefficient $\rho_{L,Y}$ remain similar across both datasets. These results occur because $\rho_{L,Y}$ quantifies the strength of the linear relationship between the predicted outputs and the true labels, rather than the exact categorical match between them. In other words, LiExNet achieves lower accuracies on FER2013 due to uncontrolled image acquisition conditions. However, it exhibits consistent performance, with the predicted outputs maintaining a strong linear relationship with the true labels and, in most cases, showing no statistically significant differences. However, it still maintains a strong linear correspondence between *L* and *Y*, even though the Wilcoxon test detects statistically significant differences in all folds of FER2013.

### 4.2. Final Performance Assessment

#### 4.2.1. Performance and Computational Cost

Table 3 presents the performance results of LiExNet, VGG16, MobileNet, ShuffleNet, and EfficientNet architectures on the CK+, KDEF, and FER2013 datasets, trained on a desktop workstation and tested on the NVIDIA Jetson device. The values in bold in Table 3 correspond to the best-performing results. VGG16, MobileNet, ShuffleNet, and EfficientNet were included in the experiments for the following reasons:

- LiExNet is inspired by the VGG16 and MobileNet architectures.
- VGG16 has been successfully used in facial expression recognition, yielding excellent results [51].
- We include the classical VGG16 model and a Lightweight-VGG16 (LVGG16) variant [52] to evaluate the baseline VGG16 architecture under different dense-layer configurations.
- We include lightweight versions of MobileNet V1 (MbNetV1), MobileNet V2 (MbNetV2), ShuffleNet (SfNet), and EfficientNet-Lite0 (ENetL), as these mobile-optimized architectures have shown competitive performance in facial expression and emotion recognition tasks [1,5,51].

**Table 3:** Accuracy and computational cost metrics obtained during testing (all computations executed in FP16 precision). Bold values denote the highest performance, and the arrows (↑, ↓) indicate the direction in which the metric shows better results.

Metric	LiExNet	VGG16	LVGG16	MbNetV1	MbNetV2	SfNet	ENetL
**Acc (CK+)**↑	**99.5%**	98.2%	97.1%	95.4%	86.8%	73.1%	77.15%
**Acc (FER2013)**↑	**79.2%**	77.4	69.84%	69.72%	65.4%	37.7%	59.3%
**Acc (KDEF)**↑	**88.2%**	83.3%	49.29%	56.25%	40.6%	65.18%	36.22%
**FPS**↑	**530.7**	11.2	12.2	104.3	195.2	193.5	102.7
**RAM (MB)**↓	**0.17**	553.4	68.38	5.3	9.361	3.72	15.76
**PRAM (MB)**↓	**2**	12.97	12.86	2.8	5.6	2.4	2.3
**Giga FLOPs**↓	0.086	30.94	0.307	10.9	0.392	**0.037**	0.258
**nParams**↓	**42,000**	138,357,544	17,926,983	1,325,829	2,340,423	976,503	4,132,010

The training of LiExNet, VGG16, LVGG16, MobileNet V1 and V2, ShuffleNet, and EfficientNet-Lite was performed using the Adam optimizer, a dynamic learning rate schedule, and categorical cross-entropy loss. The number of training epochs was adjusted according to each dataset. CK+, KDEF, and FER2013 were trained independently (without cross-dataset initialization), and no data augmentation was applied, as these settings consistently yielded the best performance in our experiments. All models were trained on a desktop workstation equipped with an AMD Ryzen 7 7700 processor, 32 GB of RAM, and an NVIDIA RTX 4060 GPU. The benchmarking and evaluation of these networks on an embedded edge device were performed using a Jetson TX2 development board (NVIDIA Corporation, Santa Clara, CA, USA). This board features a Tegra X2 System-on-Chip (SoC), an NVIDIA Pascal GPU with 256 CUDA cores, and a CPU with two Denver cores and four ARM Cortex-A57 cores. Additionally, the Jetson includes 8 GB of optimized LPDDR4 RAM with a bandwidth of 58.4 GB/s and 32 GB of eMMC 5.1 storage for data handling.

The experimental metrics evaluate both recognition performance and computational efficiency. These metrics include the average test accuracy (Acc) [53], inference speed (FPS) [54], floating-point operations per second (FLOPs) [54], model memory footprint (RAM) [55], peak inference memory (PRAM) [55], and the number of parameters (nParams) [56].

According to the values reported in Table 3, LiExNet outperforms VGG16, Simplified VGG16 (SVGG16), MobileNet variants, ShuffleNet, and EfficientNet-Lite0 across all metrics—except FLOPs, where LiExNet ranks second (slightly below ShuffleNet). However, SfNet exhibits lower accuracy across all datasets. LiExNet achieves the best FPS with a latency of 1.88 ms, the lowest RAM usage, PRAM usage, and number of parameters, while maintaining competitive computational efficiency. Moreover, since LiExNet reaches 530.7 FPS on an NVIDIA Jetson device, these results demonstrate its ability to operate in real time on embedded systems with hybrid CPU–GPU architectures.

Based on the results presented in Table 3 and the architectural description provided in Section 2, the novelty of LiExNet lies in its four-block hierarchical design, where each block integrates multi-scale feature extraction, depthwise-efficient convolutional operations, channel-wise attention, and progressive spatial reduction. This combination results in a lightweight model explicitly optimized for the extraction of expression and microexpression facial features.

**Figure 7:**
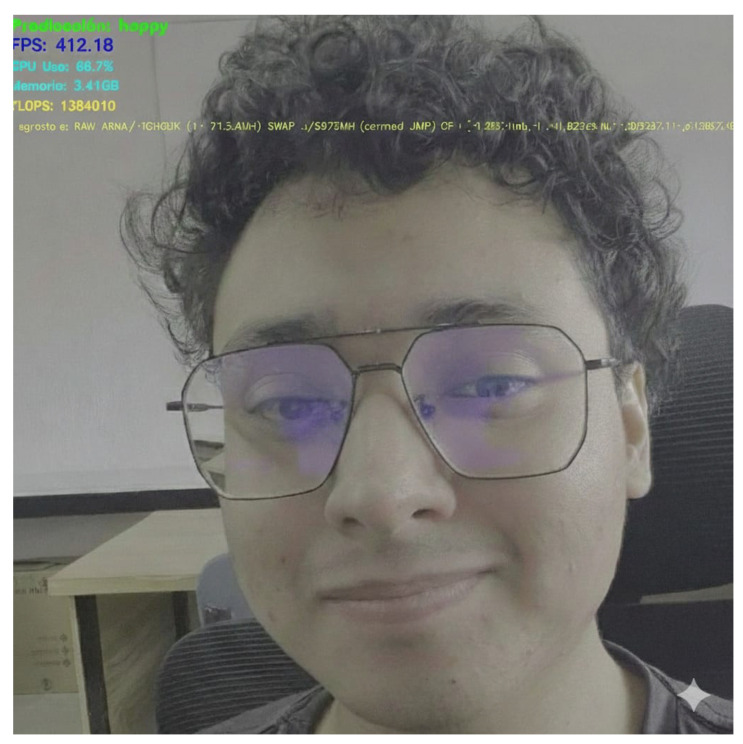
Real-time processing in Jetson TX2.

The Jetson TX2 provides up to 1.3 TFLOPs in FP16 and 8 GB of shared LPDDR4 RAM, but to run LiExNet, it must be configured with Ubuntu 18.04 LTS, Jetson Linux R32.7.4 (JetPack 4.6.4), CUDA 10.2, cuDNN 8.2.1, and TensorRT 8.2.1. In practical terms, LiExNet requires only 86 MFLOPs per inference and a peak inference memory of approximately 2 MB, which means that the model and its activations use far less than 1% of the available RAM and only a small fraction (approximately 3.5%) of the theoretical compute capacity, even at 530 FPS. These numbers indicate that LiExNet leaves ample headroom on the Jetson TX2 to execute several auxiliary services in parallel, such as image acquisition, pre-processing, logging, communication, or a graphical user interface, without saturating the device. The image $I{\left(m,n\right)}^{RGB}$ is acquired from a real-time camera connected to the Jetson TX2 via a GStreamer pipeline integrated into Jetson Linux. For example, Figure 7 illustrates a real-time processing example of LiExNet running on a Jetson TX2 device, executed under Jetson Linux.

LiExNet integrates into a typical edge AI pipeline and can be deployed on any operating system or AI framework. According to our experiments, LiExNet can run in real time while concurrently with other emotion-recognition processes, such as EEG-based analysis, sensor-fusion modules, facial analysis components, or user-interaction programs. Therefore, it is fully compatible with commercial mobile phone processors and 32-bit microcontrollers with AI accelerators, enabling a wide range of applications in affective computing and occupational dissonance assessment.

#### 4.2.2. Emotion Classification Performance

**Figure 8:**
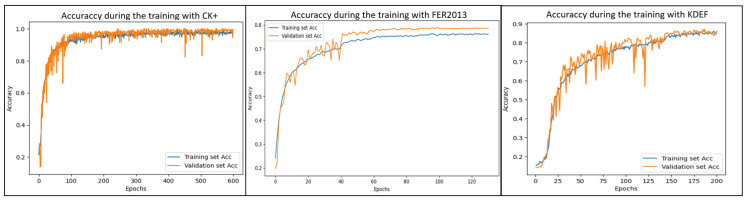
Training process generated by LiExNet with CK+, KDEF, and FER2013.

Figure 8 illustrates the accuracy trends over the training epochs for LiExNet using the CK+, KDEF, and FER2013 datasets. The following observations can be made:

- In the CK+ dataset, LiExNet reaches an accuracy of nearly 100% starting from epoch 150.
- In the FER2013 dataset, the model achieves an accuracy of approximately 80% by epoch 80.
- In the KDEF dataset, LiExNet obtains an accuracy of almost 90% by epoch 200.

In addition, the epoch-wise accuracy achieved by LiExNet on the CK+, KDEF, and FER2013 datasets is consistent between the training and test sets, indicating that LiExNet generalizes well across all three datasets.

**Figure 9:**
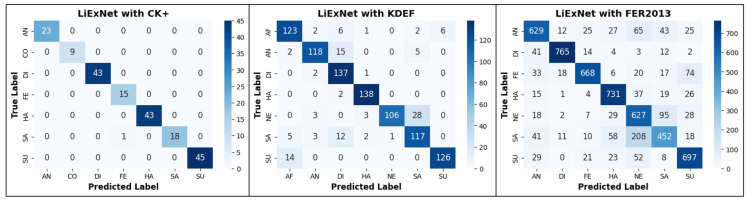
Confusion matrices generated by LiExNet with CK+, KDEF, and FER2013.

Figure 9 shows the confusion matrices obtained during the testing experiments. Regarding the CK+ dataset, nearly all emotion classes are classified correctly. Only one sample of the Sadness class is misclassified as Fear.

However, the confusion matrices of KDEF and FER2013 are more complex to interpret. Therefore, Table 4 and Table 5 were created to present the confusion matrix values normalized with respect to the total number of images in each class *l*. These normalized metrics are defined as the class-based recall ($R_{\mathrm{class}}$) and the false-negative rate per class ($F_{\mathrm{class}}$). $R_{\mathrm{class}}$ is reported along the main diagonal of the confusion matrix and is defined as $R_{class}=tp_{l}/\left(tp_{l}+fn_{l}\right)$, where $tp_{l}$ denotes the true positives for class *l*, and $fn_{l}$ represents the samples of class *l* that were misclassified into other classes. $F_{\mathrm{class}}$ corresponds to the off-diagonal elements, which represent misclassifications expressed as percentages, and is defined as $F_{\mathrm{class}}=fn_{l}/\left(tp_{l}+fn_{l}\right)$. Based on the results from the KDEF dataset presented in Table 4, several conclusions can be drawn:

- HA achieves the highest performance, where $R_{\mathrm{class}}=98.5\%$ and only 1.43% are $F_{\mathrm{class}}$ samples misclassified as DI.
- DI reaches $R_{\mathrm{class}}=97.8\%$ and only 2.14% are $F_{\mathrm{class}}$ samples misclassified as HA and AN.
- SU achieves $R_{\mathrm{class}}=90\%$ and $F_{\mathrm{class}}=10\%$, which are SU samples confused with AF.
- AF achieves $R_{\mathrm{class}}=87.9\%$ and $F_{\mathrm{class}}=12.1\%$. Among these errors, 8.6% are AF samples confused with DI and SU.
- AN achieves $R_{\mathrm{class}}=84.3\%$ and $F_{\mathrm{class}}=15.7\%$. Of these, 10.7% are AN samples confused with DI and 3.6% with SA.
- SA reaches $R_{\mathrm{class}}=83.6\%$ and $F_{\mathrm{class}}=16.4\%$. Among these, 8.6% are SA samples confused with DI and 3.6% with AF.
- NE exhibits the lowest performance, with $R_{\mathrm{class}}=75.7\%$ and $F_{\mathrm{class}}=24.3\%$. Notably, 20% of these errors are NE samples confused with SA.
- The average $R_{\mathrm{class}}$ across all classes is 88.2%, with a standard deviation of 8.1%. Therefore, HA, DI, and SU perform above the mean, and AF remains close to it, whereas AN, SA, and NE fall below.

**Table 4:** $R_{\mathrm{class}}$ and $F_{\mathrm{class}}$ of LiExNet on the KDEF dataset. The values in bold correspond to the $R_{\mathrm{class}}$.

	AF	AN	DI	HA	NE	SA	SU
AF	**87.86%**	1.43%	4.29%	0.71%	0.00%	1.43%	4.29%
AN	1.43%	**84.29%**	10.71%	0.00%	0.00%	3.57%	0.00%
DI	0.00%	1.43%	**97.86%**	0.71%	0.00%	0.00%	0.00%
HA	0.00%	0.00%	1.43%	**98.57%**	0.00%	0.00%	0.00%
NE	0.00%	2.14%	0.00%	2.14%	**75.71%**	20.00%	0.00%
SA	3.57%	2.14%	8.57%	1.43%	0.71%	**83.57%**	0.00%
SU	10.00%	0.00%	0.00%	0.00%	0.00%	0.00%	**90.00%**

**Table 5:** $R_{\mathrm{class}}$ and $F_{\mathrm{class}}$ of LiExNet on the FER2013 dataset. The values in bold correspond to the $R_{\mathrm{class}}$.

	AN	DI	FE	HA	NE	SA	SU
AN	**76.15%**	1.45%	3.03%	3.27%	7.87%	5.21%	3.03%
DI	4.88%	**90.96%**	1.66%	0.48%	0.36%	1.43%	0.24%
FE	3.95%	2.15%	**79.90%**	0.72%	2.39%	2.03%	8.85%
HA	1.80%	0.12%	0.48%	**87.76%**	4.44%	2.28%	3.12%
NE	2.23%	0.25%	0.87%	3.60%	**77.79%**	11.79%	3.47%
SA	5.14%	1.38%	1.25%	7.27%	26.07%	**56.64%**	2.26%
SU	3.49%	0.00%	2.53%	2.77%	6.27%	0.96%	**83.98%**

Table 5 shows the $R_{class}$ values and error percentages of LiExNet evaluated on the FER2013 dataset. Based on these results, several observations can be made:

- DI achieves the highest performance, with $R_{\mathrm{class}}=90.96\%$ and $F_{\mathrm{class}}=9.04\%$, which are DI samples mainly misclassified as AN.
- HA reaches $R_{\mathrm{class}}=87.76\%$ and $F_{\mathrm{class}}=12.24\%$. The $F_{\mathrm{class}}$ samples are HA mostly confused with NE.
- SU achieves $R_{\mathrm{class}}=83.98\%$ and $F_{\mathrm{class}}=16.02\%$, which are SU samples mainly misclassified as NE and SA.
- FE reaches $R_{\mathrm{class}}=79.90\%$ and $F_{\mathrm{class}}=20.10\%$. The $F_{\mathrm{class}}$ samples are FE mainly confused with SU and AN.
- NE obtains $R_{\mathrm{class}}=77.79\%$ and $F_{\mathrm{class}}=22.21\%$, which are NE samples misclassified, mostly as SA, SU, and HA.
- AN achieves $R_{\mathrm{class}}=76.15\%$ and $F_{\mathrm{class}}=23.85\%$, which are AN samples misclassified, mainly as NE, SA, and HA.
- SA exhibits the lowest performance, with $R_{\mathrm{class}}=56.64\%$ correctly classified and 43.34% of SA samples are $F_{\mathrm{class}}$. Among these errors, 26.07% correspond to confusions with NE, and notable confusion also occurs with HA and AN.
- The average $R_{\mathrm{class}}$ across all classes is 79.0%, with a standard deviation of 11.2%. Therefore, DI, HA, and SU perform above the mean, and FE is close to it, whereas AN, SA, and NE fall below.

Based on the results obtained across the three datasets, the following conclusions can be drawn: the emotions HA, DI, and SU consistently achieve the highest recognition performance. AF and FE (which correspond to the same emotion category) exhibit performance levels close to the average value of $R_{\mathrm{class}}$ in each dataset. In contrast, NE, SA, and AN show comparatively lower recognition rates.

Another factor that influences LiExNet’s performance across CK+, KDEF, and FER2013 is the manner in which the images were acquired. The CK+ dataset achieves results above 99% because all images were captured in frontal view under strictly controlled acquisition conditions. The performance decreases to 88% in KDEF, since—although captured in controlled environments—the faces exhibit variations in head orientation with respect to the camera. FER2013 yields the lowest performance, as its images were collected from the Internet and therefore present unconstrained conditions, including variations in pose, illumination, occlusions, and imaging quality.

### 4.3. Grad-CAM Analysis

**Figure 10:**
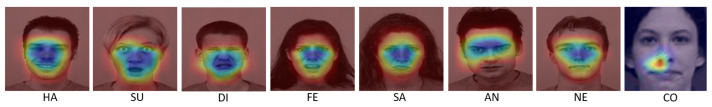
GRAD-CAM examples per class for the emotions analyzed using CK+, KDEF, and FER2013.

A Grad-CAM analysis [57] was conducted to identify the facial regions used by LiExNet for emotion discrimination. For this purpose, Grad-CAM activation maps were projected onto each facial image using the feature maps extracted from the max-pooling operation of layer l=16, as illustrated in Figure 10. A total of 63 test images were evaluated (21 from each dataset), including 54 images corresponding to Ekman’s basic emotions (9 per emotion), six from the NE class, and three from the CO class.

The visual evidence provided by Grad-CAM reveals two types of discriminative facial patterns utilized by LiExNet: expressions and microexpressions. Expressions correspond to visible emotional manifestations produced by voluntary or involuntary muscle activations, commonly referred to as Action Units (AUs) within the Facial Action Coding System (FACS). In contrast, microexpressions are brief, involuntary, and universal emotional responses that leave transient traces. In static images, these typically appear as subtle or residual Action units based on muscle activations (rAUs), which are often associated with atypical high-spatial-frequency patterns or localized facial tension [58,59,60,61,62].

Table 6 summarizes the common AU-based expressions, rAUs, and spatial-frequency microexpressions identified from the 63 analyzed Grad-CAM images, while Figure 10 exemplifies the typical activation patterns found across the different emotion categories. Emotions with the highest $R_{\mathrm{class}}$ values, such as HA, SU, and DI, exhibit strong Grad-CAM activations across multiple facial expressions and microexpressions, particularly in the central facial region. The emotions FE and AF show limited activation in the eyebrow area, but strong activation in microexpressions near the eyes and in the nasojugal region. SA and AN display Grad-CAM activations in the eyebrows, the elevated infraorbital region, and the nasojugal region. However, their expressions and microexpressions are highly similar. Finally, NE shows no consistent expression or microexpression patterns.

These findings are consistent with the theory proposed by Ekman and Friesen, which holds that positive emotions such as happiness and surprise show distinctive, highly detectable facial features with strong activation of specific expressions and microexpressions. In contrast, negative emotions—particularly sadness, fear, and anger—tend to produce more subtle and overlapping muscle activations, increasing the likelihood of misclassification [58,61,62]. Moreover, according to [59,60], the NE class has the lowest $R_{class}$ because neutral facial expressions vary widely among individuals and are strongly influenced by personality traits, thereby increasing intra-class variability.

From the 63 Grad-CAM images analyzed, the most prominent confusion occurs between the SA and NE classes. This confusion arises because SA samples typically elicit strong activations across the T-zone of the face, driven by characteristic eyebrow expressions, the infraorbital region, and nasojugal microexpressions. In contrast, NE samples occasionally produce T-zone activations because the third feature-extraction block (layers l=9,…,12) detects this region. However, the fourth block does not capture distinctive expressions or microexpressions. As a result, the T-zone activations remain unchanged across layers *l* = 12–16. This leads SA and NE to exhibit highly similar activation patterns, explaining the confusion between these two categories.

### 4.4. Liexnet Ablation Study

**Figure 11:**
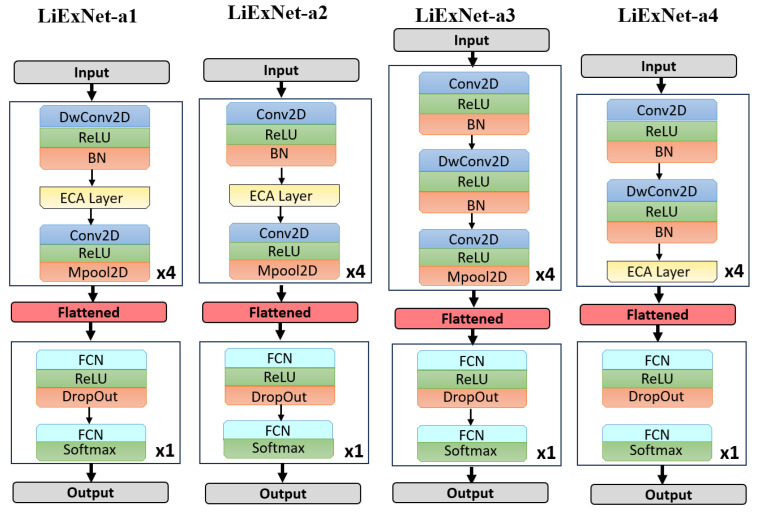
Ablation experiments.

An ablation study was performed to assess the impact of each architectural component in LiExNet. Four network variants, shown in Figure 11, were evaluated by selectively removing layers from the feature extraction blocks:

LiExNet-a1. This variant eliminates layers l={1,5,9,13}, which consist of standard convolutional layers (as described in Equation (1)) followed by batch normalization.
LiExNet-a2. This variant removes layers l={2,6,10,14}, which correspond to depthwise convolutional layers (as outlined in Equation (2)) followed by batch normalization.
LiExNet-a3. In this variant, the Efficient Channel Attention (ECA) layers l={3,7,11,15} are removed, effectively turning off the attention mechanism.
LiExNet-a4. This variant excludes layers l={4,8,12,16}, which corresponds to convolutional layers (as defined in Equation (1)) followed by max-pooling operations.

The ablation study was conducted using the KDEF dataset because LiExNet achieved intermediate performance on it. Results are presented in Table 7. Removing the convolutional or depthwise convolutional layers (LiExNet-a1 and LiExNet-a2) reduces the model’s feature-extraction capacity, leading to a notable drop in accuracy. Removing the ECA module (LiExNet-a3) results in decreased performance due to the absence of channel-wise attention. Eliminating the final convolutional and grouping stage (LiExNet-a4) forces the network to deliver as output the selection of channels containing relevant features, rather than the features themselves. These findings confirm that all components are essential and jointly contribute to maintaining LiExNet’s balance between performance and efficiency.

### 4.5. Comparison of LiExNet with Other Methods

Table 8 presents a comparison of the accuracy of LiExNet with other state-of-the-art FER methods that classify emotions into six or seven categories. The table reports the dataset used for evaluation (CK+, KDEF, or FER2013), the total number of emotions measured and evaluated by each method (Emotions), and whether the method is suitable for real-time applications (Real-time). More precisely, the total number of emotions evaluated by each method is not determined by the number of classes that the model classifies, but rather by the combined set of native emotion categories that the model measures across the datasets (CK+, KDEF, and FER2013).

Based on the results presented in Table 8, the following observations can be made:

- LiExNet has the best accuracy compared to the real-time FER methods on the CK+ and KDEF datasets, and the second-highest accuracy on FER2013.
- The method that achieves the best results for real-time FER applications on FER2013 [22] is a CNN specifically designed for emotion recognition in children with autism and evaluated exclusively on the FER2013 dataset.
- The methods reported in [33,35,36,39] achieve higher accuracy than LiExNet in one of the three datasets. However, the methods of [33,35,36] utilize CNNs with millions of parameters, which require substantial computational resources. The method of [39] is a transformer-based architecture that presents high training complexity and requires a large number of training samples. Consequently, their requirements restrict their applicability in real-time, edge-embedded, and ubiquitous sensor systems.
- The methods presented in [25,27] are evaluated using the six Ekman emotions. The methods in [36,39] and LiExNet are evaluated on the Ekman emotions together with NE and CO (i.e., the full set of emotion categories available in the datasets). The remaining methods are evaluated using only the Ekman emotions and NE.

In summary, LiExNet demonstrates superior performance among real-time FER methods and ranks among the top-performing models out of 26 state-of-the-art approaches. Its effectiveness is further evidenced by consistent generalization across datasets, as demonstrated through cross-validation, statistical significance testing, and class-level behavioral analyses across three benchmark databases. These findings highlight LiExNet as a robust and comprehensive solution for real-time emotion recognition, offering an optimal balance between accuracy and computational efficiency.

### 4.6. Experiments with EMOTION-ITCH Dataset

The confusion matrices in Figure 9 and the detailed $R_{\mathrm{class}}$ and $F_{\mathrm{class}}$ distributions shown in Table 4 and Table 5 indicate that negative emotions exhibit lower performance compared to positive emotions, as they tend to be misclassified more frequently among themselves. To address this limitation, the EMOTION-ITCH dataset groups emotions into three categories: negative, neutral, and positive. Although these categories are more general, they reduce misclassification errors and provide more consistent emotion recognition. Furthermore, the structure of the dataset enables the analysis of emotional responses in relation to habitual work-related stress. Then, we designed an experiment in which LiExNet, pre-trained on FER2013, was fine-tuned using facial expression images from the EMOTION-ITCH dataset. The fine-tuning process was performed by dividing the EMOTION-ITCH dataset into 60% (352 images) for training and 40% (234 images) for testing and for cross-validation. The objectives of this experiment were to reduce misclassification among negative emotion classes and to analyze differences in emotional responses between worker and non-worker subjects. Then, the model was initialized with the weights obtained from the FER2013 training and fine-tuned using a weighted cross-entropy loss function, defined as:

(8) $$L\left(\theta \right)=-\frac{1}{N}\sum_{i=1}^{N}h_{i}\sum_{c\in Y}{\hat{y}}_{i,c}\;\log\;\left(y_{12,d,i}\left(d|I_{i}{\left(m,n\right)}^{RGB}\right)\right),$$

where $I_{i}{\left(m,n\right)}^{RGB}$ denotes the *i*-th input image sample, *d* represents the class, ${\hat{y}}_{i,c}$ is the one-hot encoded ground truth label, and $y_{i}$ is the output of the *i*-th sample from the final layer.

The images corresponding to working subjects were assigned the following weights:

(9) $$S_{w}=\left\{\begin{array}{cc} S_{w}, & \mathrm{If}\;\mathrm{the}\;\mathrm{subject}\;\mathrm{works},\\ 1, & \mathrm{Another}\;\mathrm{case}, \end{array}\right.$$

where $S_{w}\in \left\{1,1.5,2,3,4\right\}$ is a weighting factor used to emphasize the samples from working subjects.

**Figure 12:**
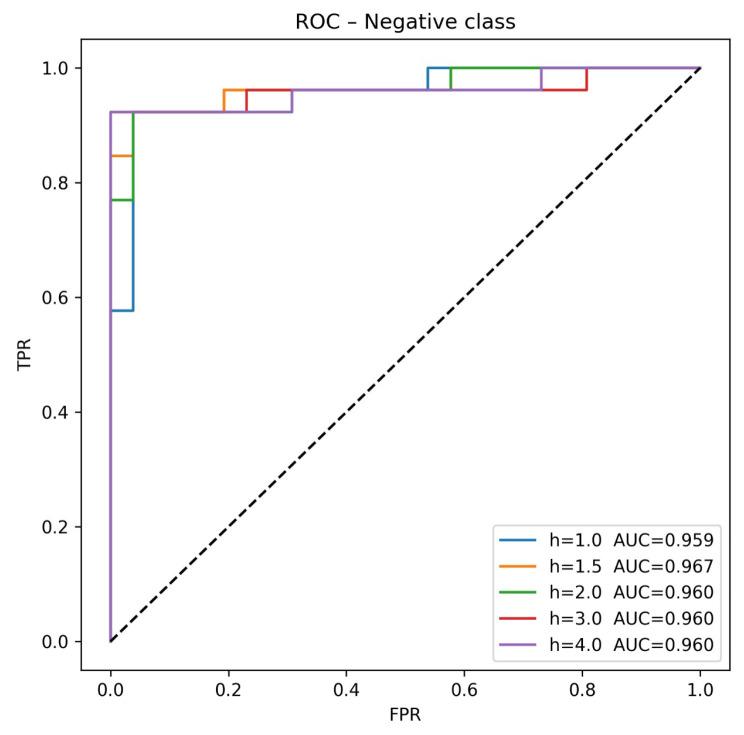
AUC with various $S_{w}$ weight adjustments. The dashed line represents the random performance.

The metrics used in this experiment were accuracy (Acc), recall for the negative class ($R_{class}$), and the Area Under the Curve (AUC). The $R_{class}$ is defined as follows:

(10) $$R_{neg}=\frac{tp_{neg}}{tp_{neg}+fn_{neg}}$$

where $tp_{\mathrm{neg}}$ refers to the number of negative emotion samples correctly classified as negative, and $fn_{\mathrm{neg}}$ refers to the number of negative emotion samples incorrectly classified as positive. The AUC is defined as follows:

(11) $$AUC=\int_{0}^{1}tpr\left(i\right)\;dfpr\left(i\right)$$

where tpr(i) and fpr(i) represent the true positive rate and false positive rate, respectively, for the *i*-th sample. Table 9 reports the results of the fine-tuning experiment:

The AUC, presented in Figure 12, can be interpreted as follows:

- A weight of $S_{w}=1.5$ yields the best AUC.
- A weight of $S_{w}=2$ generates a slight improvement in AUC compared to $S_{w}=1$, and outperforms $S_{w}=1.5$ in terms of Acc and $R_{class}$.
- A weight of $S_{w}=3$ achieves the best values for Acc and $R_{\mathrm{class}}$.
- A weight of $S_{w}=4$ yields the same AUC as $S_{w}=2$ and $S_{w}=3$. However, both Acc and $R_{\mathrm{class}}$ decrease significantly.

Based on these points, it can be observed that information about whether subjects work or not contributes to model performance when $S_{w}\in \left\{1.5,2,3\right\}$. Specifically, with $S_{w}=1.5$, the model exhibits improved discrimination capability between classes, particularly for the negative class, across all possible thresholds. In contrast, h=3 yields the best classification accuracy. The weight of $S_{w}=4$ suggests a potential overemphasis on the working subjects. These findings indicate that including the weight factor $S_{w}$ improves inter-class separability, as prior knowledge of the affective state (habitual work-related stress or relaxed) helps extract more discriminative features from facial expressions and microexpressions, thereby enhancing emotion recognition.

**Figure 13:**
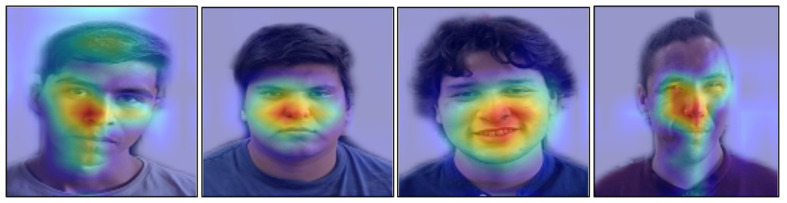
Grad-CAM visualizations for two subjects with working conditions and $S_{w}=2$.

A GRAD-CAM experiment was applied to visualize the facial regions that LiExNet focuses on during emotion recognition, thereby improving interpretability. According to the Grad-CAM analysis, LiExNet effectively suppresses background information and concentrates on the salient facial regions described in Table 6. The model primarily attends to the eyes, nose, cheeks, and lips, as well as subtle microexpressions located around the nose and below the eyes, as illustrated in Figure 13.

To verify the consistency of the results with EMOTION-ITCH, we perform 5-fold cross-validation and a statistical significance analysis using $\rho_{L,Y}$ and the Wilcoxon test, with LiExNet trained via fine-tuning and $S_{w}=3$. Table 10 shows that the folds yield very similar results, all of which are close to 100%. The values of $\rho_{L,Y}$ and $h_{L,Y}$ indicate that the label set *L* and the prediction set *Y* are nearly identical across all folds.

## 5. Conclusions

This paper presents LiExNet, a deep neural network with strong generalization and top accuracy across multiple datasets, designed for facial expression-based emotion recognition with low computational complexity, making it suitable for real-time deployment in embedded and sensor-driven ubiquitous systems. The model was evaluated using the CK+, KDEF, FER2013, and EMOTION-ITCH datasets. CK+, KDEF, and FER2013 are well-established and widely used in the literature. EMOTION-ITCH is a custom multimodal dataset specifically developed to investigate the relationship between emotion recognition and habitual work-related stress under realistic acquisition conditions.

Cross-validation experiments indicate that LiExNet achieves accuracies of 99.36%±1.2 on CK+, 88.3%±0.75 on KDEF, and 79.2%±0.9 on FER2013. The final test results report accuracies of 99.5% on CK+, 79.2% on FER2013, and 88.2% on KDEF. Statistical significance analyses further demonstrate that LiExNet learns and generalizes consistently across all datasets. Overall, these differences are expected, not because of the LiExNet architecture, but because CK+ contains frontal faces acquired under controlled conditions, KDEF includes faces captured from multiple angles under controlled settings, and FER2013 contains facial images collected in unconstrained, highly variable real-world environments.

According to the emotion classification analysis conducted on the KDEF and FER2013 datasets, LiExNet achieves class-based recall ($R_{\mathrm{class}}$) values of 99.3%±1.9 for CK+, 78.8%±10.7 for FER2013, and 86.5%±6.9 for KDEF. The highest-performing emotions are HA, SU, and DI, whereas AF and FE exhibit intermediate behavior. The lowest-performing categories are SA, AN, and NE. The Grad-CAM analysis suggests that this behavior arises because HA, SU, and DI exhibit a wide range of expressions and microexpressions, whereas AF, FE, SA, and AN share similar expressions and microexpressions patterns. In contrast, the NE category lacks consistent facial features that can be reliably used for classification.

Sadness and neutrality yield slightly lower performance, as these emotions are occasionally confused with one another. Similarly, anger and fear show reduced accuracy because they frequently overlap with other categories. To address these challenges, a fine-tuning experiment was conducted using the EMOTION-ITCH dataset, which groups emotions into positive, negative, and neutral classes to improve robustness under real-world acquisition conditions. Results show that LiExNet attains an accuracy of 90.38%, which increases to 96% when prior knowledge of the subject’s affective state is incorporated. These findings demonstrate that LiExNet is effective for affective computing applications in sensor-based and embedded environments, particularly in occupational scenarios where emotional dissonance and stress monitoring are relevant.

LiExNet has a computational cost of 86 MFLOPs and an architecture comprising approximately 42,000 parameters, a 0.17 MB model memory footprint, and a peak inference memory of 2 MB. This low architectural complexity enables real-time inference on CPU–GPU embedded platforms commonly used in sensor-driven systems. Both a desktop workstation and an NVIDIA Jetson TX2 achieved instantaneous, low-latency processing. Moreover, LiExNet requires only a minimal fraction of the computational and memory resources available in embedded devices, allowing it to operate concurrently with other sensing, processing, or decision-making modules within edge-AI architectures.

Given its balance between performance and computational efficiency, LiExNet stands among the most suitable real-time FER models for deployment in embedded and ubiquitous environments, including mobile devices, smartphones, and affective computing systems. Future work will focus on two main directions. First, we aim to explore strategies to train LiExNet to achieve strong performance on facial images captured under uncontrolled conditions and with faces in varying poses. Second, it is necessary to integrate LiExNet into an application programming interface (API) for edge-computing platforms, expanding its applicability to sensor-driven affective computing, emotional dissonance monitoring in occupational environments, automotive driver-assistance systems, and human–machine interaction scenarios.

## Figures and Tables

**Table 1:** 5-fold cross-validation accuracy of LiExNet on the CK+, FER2013, and KDEF datasets, represented by the per-fold results.

Dataset	*k* = 1	*k* = 2	*k* = 3	*k* = 4	*k* = 5	μ±σ
CK+	99.5%	100%	100%	97.4%	100%	99.3% ± 1.2
KDEF	89.1%	87.2%	87.5%	87.8%	88.1%	88.3% ± 0.75
FER2013	79.7%	79.78%	79.24%	77.6%	79.72%	79.2% ± 0.9

**Table 2:** Results of $\rho_{l,y}$, $h_{l,y}$, and $p_{l,y}$ obtained for different *k*-folds.

	k=1	k=2	k=3	k=4	k=5
$\rho_{l,y}$ with CK+	0.98	1	1	0.97	1
$h_{l,y}$ with CK+	0	0	0	0	0
$p_{l,y}$ with CK+	100%	100%	100%	50%	100%
$\rho_{l,y}$ with KDEF	0.81	0.80	0.81	0.81	0.78
$h_{l,y}$ with KDEF	0	1	1	0	0
$p_{l,y}$ with KDEF	11%	2%	1%	28%	5.5%
$\rho_{l,y}$ with FER2013	0.801	0.803	0.812	0.781	0.801
$h_{l,y}$ with FER2013	1	1	1	1	1
$p_{l,y}$ with FER2013	0.3%	0.3%	2%	1%	2%

**Table 6:** Facial expressions and microexpressions by emotion.

Emotion	Expressions	Microexpressions
HA	Raised cheeks.	rAUs with elevated lip corners and high-frequency patterns caused by wrinkles under the eyes.
SU	Wrinkled nose	Upper lip raised and rAUs appearing in the central facial region.
DI	Widening of the nostrils and upper lip raised.	Fine lines in the nasolabial fold.
FE/AF	Eyebrows forming an inverted triangle shape.	rAUs in the infraorbital region and rAUs in the nasojugal groove.
SA	Eyebrows forming an inverted triangle shape and elevated infraorbital region.	rAUs in the nasojugal groove.
AN	Eyebrows pulled together and elevated infraorbital region.	High-frequency patterns caused by wrinkles between the nose and eyes.
CO	Asymmetry in the position of eyes and lips.	rAUs in the nasolabial region.
NE	No muscle activation.	No microexpressions.

**Table 7:** Accuracies obtained from ablation study.

LiExNet-a1	LiExNet-a2	LiExNet-a3	LiExNet-a4
0.6446	0.4873	0.4822	0.4416

**Table 8:** Comparison of LiExNet performance with other methods across datasets. This comparison includes the number and type of emotion classes used.

Method	CK+	KDEF	FER2013	Emotions	Real-Time
Ullah et al. [25]	91.42%	–	–	6	No
Jain et al. [27]	93.24%	–	–	6	No
Elsheikh et al. [26]	99.29%	–	–	7	No
Sun et al. [28]	98.38%	–	–	7	No
Sen et al. [37]	91.11%	–	–	7	No
Kumar et al. [38]	98.9%	–	–	7	No
Yang et al. [14]	92.4%	–	–	7	Yes
Khattak et al. [15]	95.65%	–	–	7	Yes
Chowdary et al. [16]	98.5%	–	–	7	Yes
Minaee et al. [17]	98%	–	–	7	Yes
Happy et al. [18]	94.09%	–	–	7	Yes
Gupta et al. [19]	92.32%	–	–	7	Yes
Akhand et al. [35]	–	96.51%	–	7	No
Hussain et al. [24]	–	88%	–	7	Yes
Haider et al. [34]	–	–	74.64%	7	No
Zhang et al. [33]	–	–	88.56%	7	No
Amal et al. [20]	–	–	75.1%	7	Yes
Shah et al. [21]	–	–	63.2%	7	Yes
Sholikah et al. [22]	–	–	91%	7	Yes
Shehu et al. [23]	97.86%	85%	–	7	Yes
Mehendale et al. [29]	94%	–	73%	7	No
Sarvakar et al. [30]	96%	–	71%	7	No
Li et al. [31]	98.9%	–	75.82%	7	No
Li et al. [32]	94.1%	–	72.35%	7	No
Khan et al. [36]	–	99.3%	78.6%	8	No
Nawaz et al. [39]	99.5%	–	98.67%	8	No
LiExNet (this work)	99.5%	88.2%	79.2%	8	Yes

The 6-emotion configuration corresponds to HA, SA, FE, AN, DI, and SU. The 7-emotion configuration corresponds to AN, DI, FE, HA, SA, SU, and NE. The 8-emotion configuration corresponds to AN, DI, FE, HA, SA, SU, NE, and CO. The methods evaluated on the FER2013 dataset and reported in this comparative study use a refined version of the dataset, as described in Section 3.3.

**Table 9:** Results of the fine-tuning of LiExNet with EMOTION-ITCH considering different weighting factors $S_{w}$.

Metric	$S_{w}=1$	$S_{w}=1.5$	$S_{w}=2$	$S_{w}=3$	$S_{w}=4$
AUC	0.959	0.967	0.960	0.960	0.960
$R_{neg}$	0.8461	0.8462	0.9231	0.9231	0.8077
Acc	90.38%	92.30%	94.23%	96.2%	90.38%

**Table 10:** Cross-validation and statistical significance results.

Folder	k=1	k=2	k=3	k=4	k=5	μ±σ
Acc	98.1	100	98.1	95.5	96.5	97.57 ± 1.78
$\rho_{L,Y}$	0.992	1	98.1	0.974	0.981	-
$h_{L,Y}$	0	0	0	0	0	-

## Data Availability

The original data presented in the study are openly available in EMOTION-ITCH, which is available at https://sites.google.com/view/laboratoriodps/download?authuser=0 (updated on 25 November 2025).
